# Personalized Offline and Pseudo-Online BCI Models to Detect Pedaling Intent

**DOI:** 10.3389/fninf.2017.00045

**Published:** 2017-07-11

**Authors:** Marisol Rodríguez-Ugarte, Eduardo Iáñez, Mario Ortíz, Jose M. Azorín

**Affiliations:** Brain-Machine Interface Systems Lab, Systems Engineering and Automation Department, Miguel Hernández University of Elche Elche, Spain

**Keywords:** pedaling intention, pseudo-online, offline, electrode configurations, feature extraction algorithms, personalized brain-computer interfaces

## Abstract

The aim of this work was to design a personalized BCI model to detect pedaling intention through EEG signals. The approach sought to select the best among many possible BCI models for each subject. The choice was between different processing windows, feature extraction algorithms and electrode configurations. Moreover, data was analyzed offline and pseudo-online (in a way suitable for real-time applications), with a preference for the latter case. A process for selecting the best BCI model was described in detail. Results for the pseudo-online processing with the best BCI model of each subject were on average 76.7% of true positive rate, 4.94 false positives per minute and 55.1% of accuracy. The personalized BCI model approach was also found to be significantly advantageous when compared to the typical approach of using a fixed feature extraction algorithm and electrode configuration. The resulting approach could be used to more robustly interface with lower limb exoskeletons in the context of the rehabilitation of stroke patients.

## 1. Introduction

The scientific community has increasingly become more conscious of the problems suffered by people with motor disabilities, including their rehabilitation process. The use of brain-computer interfaces (BCIs) as an alternative pathway for those people who cannot move their limbs properly has been extensively studied in the literature (Dobkin, 2007; Daly and Wolpaw, 2008). Offline processing of the electroencephalography (EEG) signals can often be accurate and useful as an a posteriori tool, while a continuous processing of the signals, called here a pseudo-online analysis, may produce results of lower quality, but is much more reliable for use in active therapies that directly involve the patient's central nervous system (Daly and Wolpaw, 2008; López-Larraz et al., 2014). Indeed, exoskeletons, which are devices assisting a patient's affected limb (Hortal et al., 2015), can be combined with BCIs to improve the rehabilitation process in terms of time and quality (Pennycott et al., 2012; Rodríguez-Ugarte et al., 2016).

The basis of BCIs is to extract neural oscillations (often in the form of EEG signals), commonly known as brain waves, and translate them into commands to control a device. These waves are categorized by the frequency bands associated with the performance of some activity, and by the predominant location where they are generated (Rao, 2013). Some frequency bands are: delta (0.1–4 Hz), associated to deep sleep (Amzica and Steriade, 1998); theta (4–7 Hz), related to drowsiness (Schacter, 1977); alpha (8–15 Hz); mu (8–12 Hz), detectable in the sensorimotor cortex (Steriade, 2005); beta (16–31 Hz), detectable over the parietal and frontal lobe (Rao, 2013); and gamma (21–100 Hz).

Accurately detecting movement intent ideally involves detecting the movement before it even initiates, but may encompass both the moments previous and the initial phases of movement. In any case, the idea is to detect the movement as early as possible from the brain waves, and typically, the earlier one wishes to detect movement initiation, the more challenging it becomes. Recent studies have found two phenomena to detect movement intent: the Bereitschaftspotential (BP) (Bhagat et al., 2014; Xu et al., 2014) and event-related desynchronization/synchronization (ERD/ERS) (Bai et al., 2007; Planelles et al., 2014). BP is a motor related cortical potential (MRCP) (Jahanshahi and Hallett, 2003). Its detection usually requires averaging across many trials due to its small amplitude and low frequency, meaning that its real-time detection is typically not viable. Meanwhile, ERD/ERS are frequency fluctuations detectable in the mu and beta bands. These phenomena start about 2 s before movement onset with a decrease of the band power (ERD), followed by its increment (ERS) at about 2 s after the movement onset (Toffanin et al., 2007; Nam et al., 2011). Overall, detection of movement intent using EEG signals has been studied in subjects when performing reaching tasks, walking, hand movement or hand motor imagery (Bai et al., 2007; Ibáñez et al., 2010; Bai et al., 2011; Lew et al., 2012; López-Larraz et al., 2014; Sburlea et al., 2015). However, many of these studies suffer from only being tested offline, or from experimental setups that produce unrealistic EEG signals when compared to real-life self-initiated movement (Pfurtscheller et al., 2006; Lehtonen et al., 2008).

Outcomes of the effectiveness of BCIs on detecting an activity are often highly subject-dependent (Ang and Guan, 2013; Rohm et al., 2013), and therefore processing the data in several different ways and choosing the most effective way for each subject can be very useful in improving the results. Key factors in data processing include window selection (whether only time before movement onset, or time before and after movement onset is considered), electrode configurations and feature extraction algorithms. The usual approach involves uniformly using a fixed subject-independent electrode configuration (with associated filters) and feature extraction algorithm. On the other hand, the approach in this work is distinctive in being flexible on the choice of these parameters, so that the BCI model is better adapted to each subject.

The purpose of this work was to design a personalized BCI capable of detecting the intention of self-initiated pedaling. This included consideration of a wide array of processing algorithms for both offline and pseudo-online. To compare the results, a metric was defined, and a procedure to choose the best algorithm for each particular subject was described. More precisely, two different processing windows, with eight different electrode configurations, and with five different extraction algorithms were studied. To determine the effectiveness and reliability of the BCI, the average and variance of three important parameters were reported: the true positive rate (TPR), the false positives per minute (FP/min) and the accuracy (Acc).

Detection of asynchronous pedaling intention was contemplated in the context of stroke patients looking to improve their walking ability. The results in this work are a stepping stone toward that final goal. Thus, there was more effort invested in the analysis of pseudo-online processing, due to its relevance in active therapies involving patients (e.g., via exoskeletons). Having said that, for now, only healthy subjects were considered. Pedaling, which is a complex motor task, was chosen over walking, because it may represent an important intermediate step in patient recovery before attempting gait. Additionally, it permits a more controlled experimental setting of the lower limbs, which for example avoids artifacts such as head movements.

## 2. Materials and methods

### 2.1. Subjects

Five healthy subjects between 24 and 35 years old (3 males and 2 females, 28.6 ± 4.2 years), all right footed, took part in this experiment. The subjects did not have any known neurological diseases and all of them gave written informed consent according to the Helsinki declaration. The Ethics Committee of the Office for Project Evaluations (Oficina Evaluadora de Proyectos: OEP) of the Miguel Hernández University of Elche (Spain) approved the study.

### 2.2. Test description

**Figure 1:**
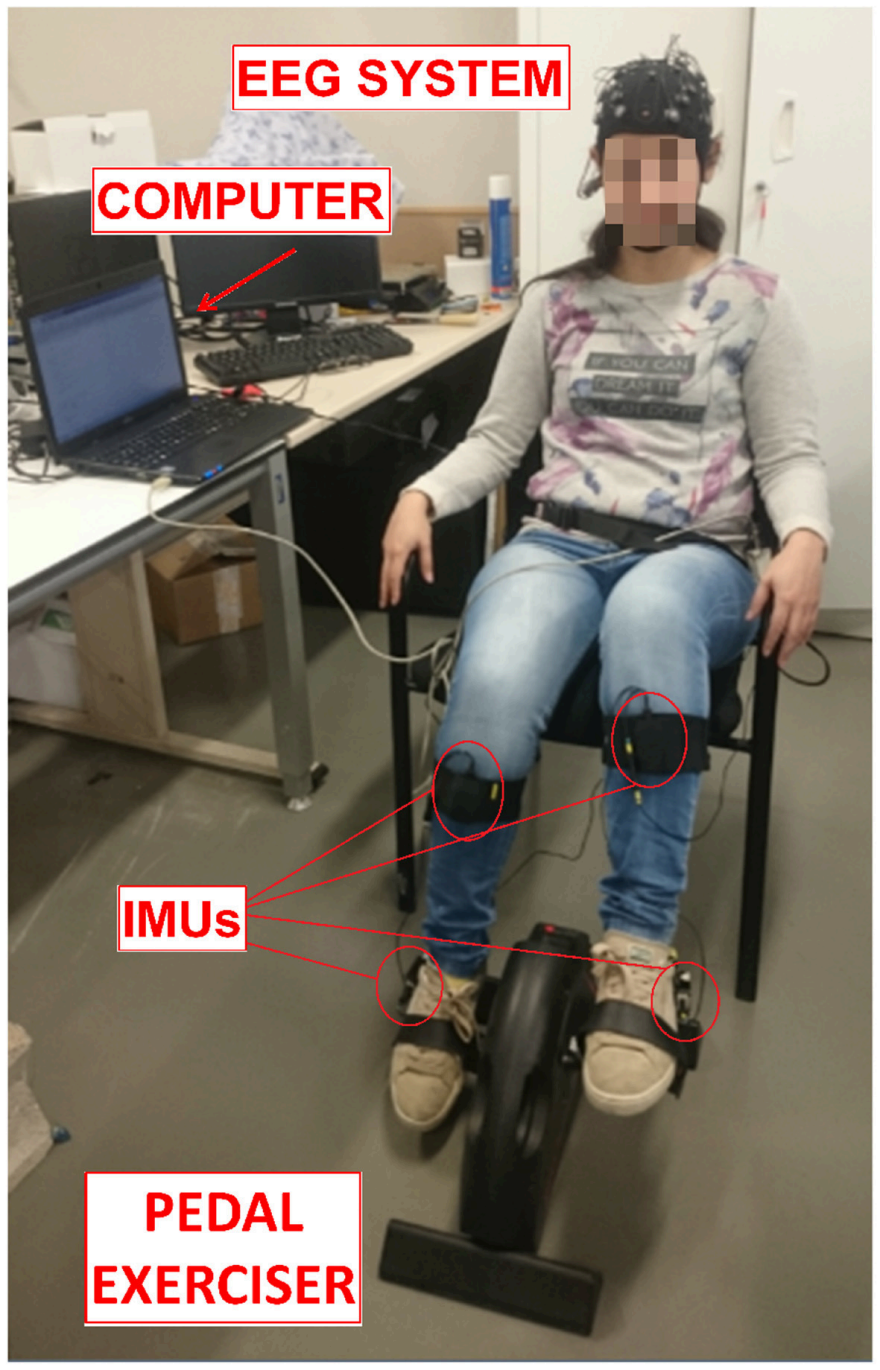
Experiment setup. Subjects sat at a comfortable distance to pedal in a pedal exerciser wearing two IMUs per leg and an EEG reader. Both systems were connected to a computer to process the signal. The participant in the picture gave written informed consent to publish the image.

**Figure 2:**
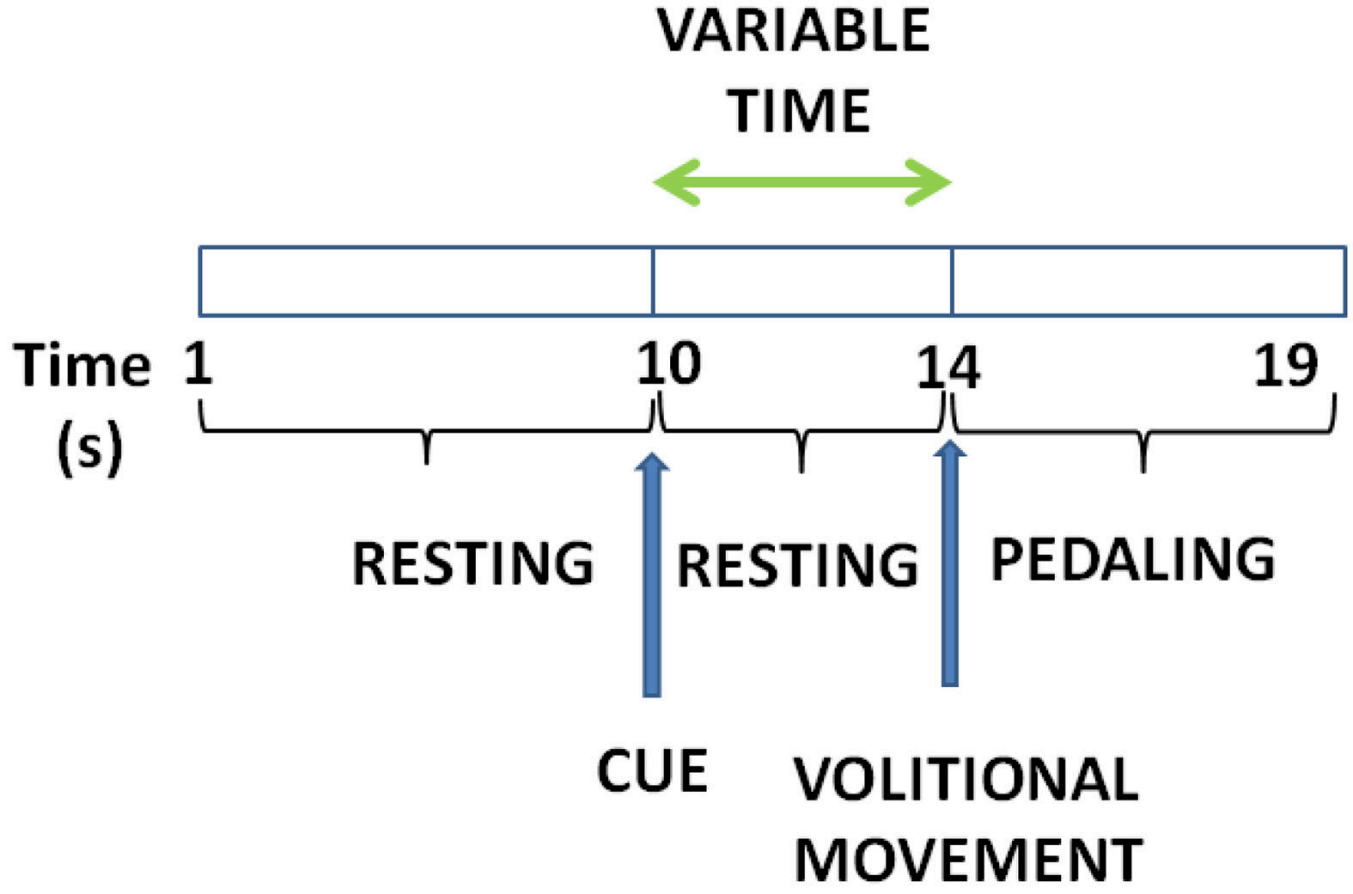
Experiment protocol: Subjects remain for about 10 s still followed by a cue to start pedaling for about 5 s. Time between the cue and the pedaling start is at least 3 s.

The experiment consisted on pedaling and resting during periods of time in which EEG and motion signals were recorded. Subjects were sat at a comfortable distance from a pedal exerciser, as shown in Figure 1. Each subject performed one session, which was composed of 16 trials. Each trial consisted of 5 pedaling cycles, with each cycle defined as: 10 s of resting, followed by a cue from the experimenter indicating the subject to initiate pedaling at their own volition for about 5 s. Before starting the experiment, subjects were told to wait without counting a minimum of 3 s between the cue and the pedaling movement. This requirement was specified in order to avoid the influence of cue presentation in the EEG signals. If this period was not fulfilled, the trial was discarded. Figure 2 shows a sketch of the protocol.

### 2.3. BCI design

A BCI aiming to detect pedaling intent through EEG signals was designed by searching for ERD/ERS in the mu and beta bands. To achieve this, five different feature extraction algorithms were considered. In addition, each algorithm was applied using two different types of processing windows: those with only time before movement onset, and those with time before and after movement onset. Furthermore, each combination of window type and algorithm was tested with eight different electrode configurations. Then, all this data was utilized to select a personalized BCI for each subject. This will be explained in more detail throughout this section.

Data was divided in two types: training and test data. Both types follow the same process: signal acquisition, data selection, preprocessing, channel selection, and feature extraction. The features and their corresponding class of training data were used to create the model. Then, that model was used to classify the features extracted from the test data. More details will follow later.

In terms of software, a MATLAB (MathWorks Inc., Massachusetts, United States) platform was developed to record, process and classify the EEG signals. Test data was processed using offline and pseudo-online analyses. The offline analysis consists of recording the EEG signals and then, without the acquisition hardware, loading and analyzing them. As will be observed later, classification and processing of data analyzed offline requires the knowledge of the full signal beforehand (e.g., use of cross validation and majority vote algorithms). On the other hand, the pseudo-online analysis bears some similarities with the offline analysis in the local processing of the data, but crucially differs in that the EEG signals are treated as if they were to be processed in real-time. Therefore, only the data before a given time is used for classification, and data selection requiring prior knowledge of the movement onset is impossible with this type of analysis. Hence, the pseudo-online analysis is more challenging to design and implement than the offline analysis, but has the advantage of having potential use in real-time activities, such as interfacing with exoskeletons. The word *pseudo* is used here to clarify that in this work the EEG data was not processed in real-time during the experiments themselves, but instead was collected and a posteriori was treated as such. This was done to test the different pseudo-online schemes, so that in the future a real-time processing of data is successfully achieved.

#### 2.3.1. Signal acquisition

The Enobio 32 EEG system (Neuroelectrics, Barcelona, Spain) was used to extract the signals from the brain. It is a wireless device with 32 electrodes based on the International 10-10 system (P7, P4, CZ, PZ, P3, P8, O1, O2, C2, F8, C4, F4, FP2, FZ, C3, F3, FP1, C1, F7, OZ, PO4, FC6, FC2, AF4, CP6, CP2, CP1, CP5, FC1, FC5, AF3, PO3) and with two reference electrodes (CMS and DRL). The reference electrodes were located on each subject's earlobe with the help of an earclip. Signals were acquired at a sampling frequency rate of 500 Hz. This system is shown in Figure 1.

Furthermore, to verify the reliability of the BCI system, its output was compared to the Tech MCS system's output (Technaid S.L., Spain). This wireless device is based on inertial measurement units (IMUs). Each IMU is composed by three micro sensors: a 3D gyroscope, a 3D magnetometer and a 3D accelerometer. Nineteen parameters are registered by each IMU, but only the gyroscope in X was utilized to detect when a real start of the pedaling movement was produced. Data was registered at a frequency of 20 Hz through a HUB connected to the USB port of the PC. Subjects wore two IMUs per leg: one located on the external part of the ankle and the other one located on the tibialis anterior, as can be appreciated in Figure 1. It should be noted that there are “cheaper” alternatives to measuring angular velocities which do not involve IMUs, but ultimately equipment availability was the deciding factor.

#### 2.3.2. Data selection

The angular velocities associated to the two IMUs in each leg were averaged, and the real start was defined when both leg averages exceeded a threshold. Meanwhile, 32 EEG signals where acquired and, part of this data was selected.

**Figure 3:**
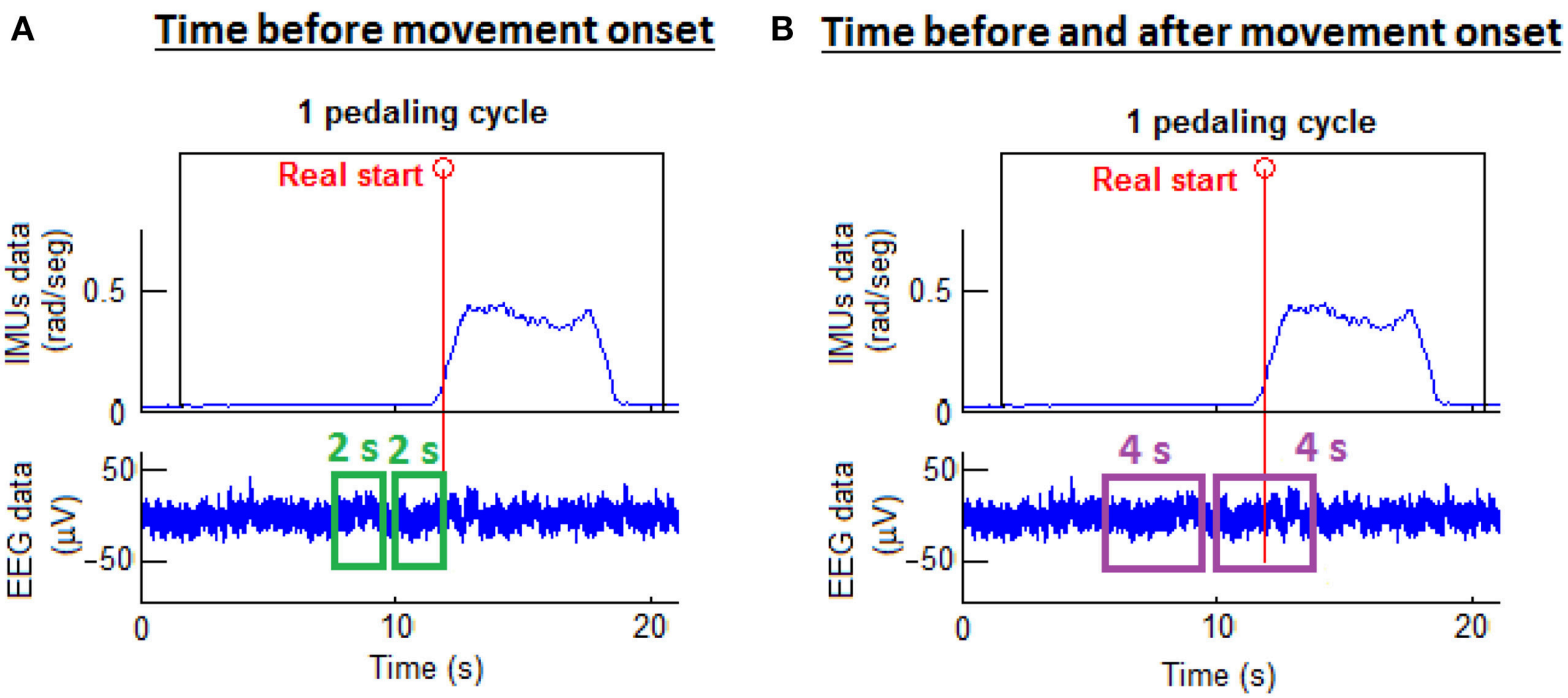
Processing window types. On the left **(A)**, the start class window selection was 2 s before the movement onset. On the right **(B)**, the start class was 2 s before and 2 s after the movement onset.

As reported in Toffanin et al. (2007), the potential generated by movement intention appears 2 s before the movement onset and it lasts until around 2 s after the onset. Hence, two types of processing windows were studied: 2 and 4 s processing windows. In addition, data was separated in two classes: rest and start. These two classes were defined according to the real start and the processing window selected. For 4 s processing windows, the start class window was defined from 2 s before to 2 s after the real start was produced. For 2 s processing windows, the start class window was defined from 2 s before up to the real start. The rest class window was chosen to be the same duration as the corresponding start class window and was located before the start class window with a gap of 0.5 s between the two classes. Figure 3 presents these two classes with the two different types of processing windows. The EEG training data was selected from these class windows.

For the offline analysis, the window placement also determined where EEG test data was selected from, while for the pseudo-online analysis the whole pedaling cycle data was used as EEG test data. Thus, for the pseudo-online analysis, the window placement merely served as a marker to determine if a given detection was located in the start class window or not.

#### 2.3.3. Pre-processing

Preprocessing was carried out in order to improve the signal to noise ratio. Rest and start class windows were analyzed in 1 s epochs with a 200 ms shift. For each epoch a notch filter was applied to suppress the power line interference at 50 Hz. Then, a 4th order Butterworth high-pass filter with a cut-off frequency of 0.2 Hz was used to remove the direct current. Finally, a common average reference (CAR) filter was computed as in McFarland et al. (1997). This filter removed from each electrode the influence of the other ones by using the mean potential.

#### 2.3.4. Channel selection

**Figure 4:**
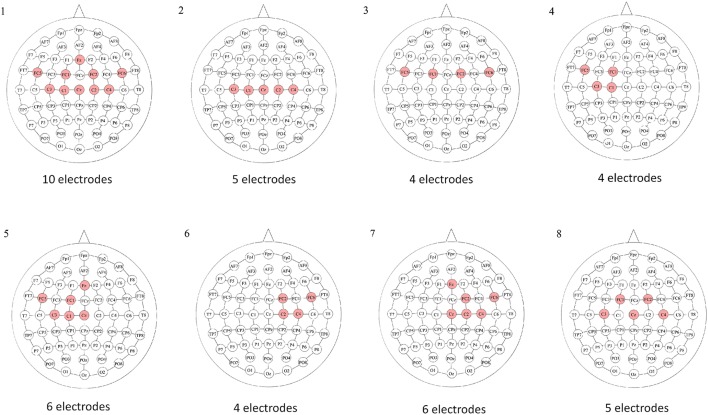
Electrode configurations based on the International 10–10 system. For each electrode configuration, the red electrodes were the ones selected. Configuration 1 focused on the motor and premotor areas; 2 focused on the motor area; 3 focused on the premotor area; 4 focused on the left side of the motor and premotor areas; 5 focused on the left side of the motor, premotor and medial areas; 6 focused on the right side of the motor and premotor areas; 7 focused on the right side of the motor, premotor and medial areas; and 8 was a reduced version of the first configuration, which focused on the motor and premotor areas.

Once the epoch was preprocessed, an electrode configuration was selected. Eight different electrode configurations (E.C.) were studied to determine which one presented better results for each subject. These are illustrated in Figure 4. The electrodes of each configuration were located on the somatosensory and motor cortex, which were the areas where most of the neural activity was expected.

#### 2.3.5. Feature extraction

For each epoch of interest, five different feature extraction algorithms were implemented in order to evaluate the optimal one for each subject:

- Algorithm A: The mean bandpower of the signal between 18 and 28 Hz was calculated for each electrode. Therefore, this method provided one feature per electrode.
- Algorithm B: Fast Fourier Transform (FFT) was applied to each electrode to evaluate the spectrum in the 0–50 Hz frequency range with 1 Hz resolution. The Euclidean norm of the resulting vector (of size 50) was then computed. Using this method, there was one feature per electrode.
- Algorithm C: This algorithm computed the mean of the power spectral density of the bands of 1–4 Hz, 8–12 Hz, and 13–28 Hz. Therefore, for each electrode there were three features.
- Algorithm D: For this method, the best frequency for each electrode, which corresponds to the potential with the highest variation between classes, was calculated. First, the power spectral density between 8 and 28 Hz with 0.5 Hz of resolution was applied. Then, rest and start class were separated and normalized. For each electrode, the frequency for which the maximum difference between classes occurred was selected and denominated as the optimal frequency. These optimal frequencies for each electrode were part of the model. Finally, the mean power spectral density of each electrode in the range of its optimal frequency ± 1 Hz was calculated. Using this method there was one feature per electrode.
- Algorithm E: FFT with a 1 Hz resolution was applied to each electrode. Then, the sum of three frequency ranges was determined for each electrode: mu (8–12 Hz), beta low (12–24 Hz) and beta high (24–30 Hz). This method provided three features for each electrode.

#### 2.3.6. Classification

A support vector machine (SVM) classifier was used to create the model and classify the data. This classifier is based in hyperplane separation by maximizing the margin between the nearest points of the different classes (Steinwart and Christmann, 2008). When combined with nonlinear kernels, the classifier is one of the most robust and often provides better outcomes than other classifiers like linear discriminant analysis (Hortal et al., 2016; Sburlea et al., 2016). In this work a radial basis function was utilized as a kernel for the SVM.

**Figure 5:**
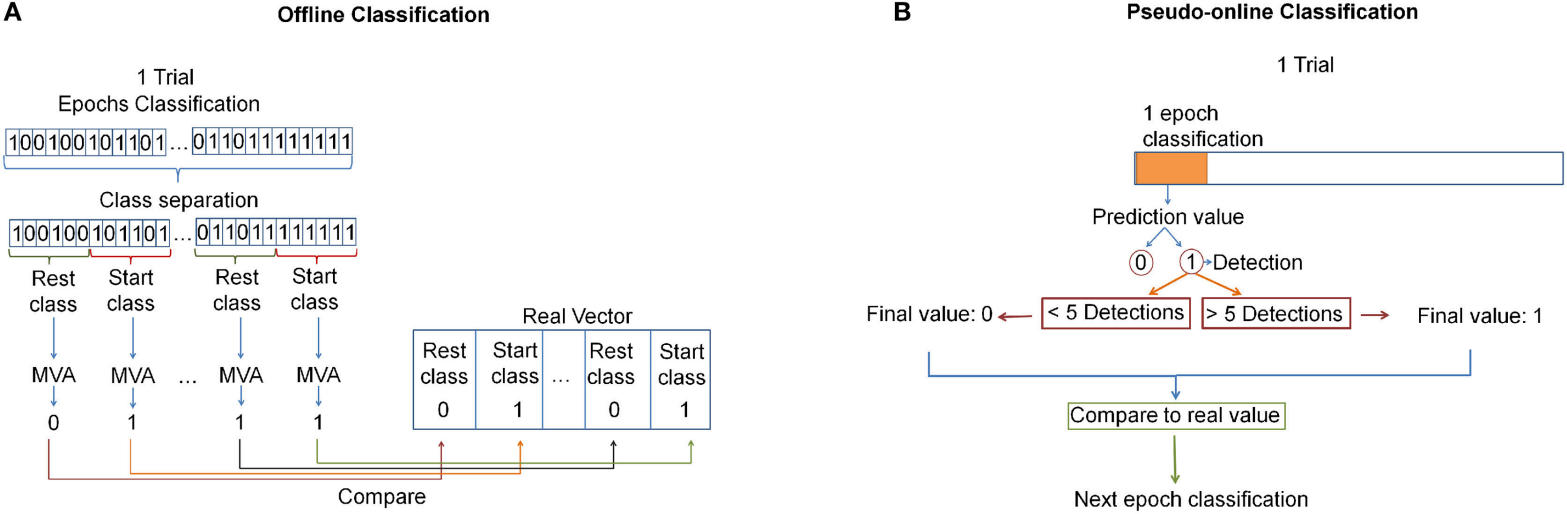
**(A)** Offline analysis: All epochs are classified. Then, a MVA is applied for each class window. Finally it is compared with the real vector. **(B)** Pseudo-online analysis: Each epoch is analyzed. A pedaling intention detection is produced after 5 successive detections. This value is compared with the real one.

For the offline analysis a cross-validation was performed: fifteen trials were used to create the model and one to test it; the process was repeated 16 times until all the trials were tested; and the results were averaged among the 16 repetitions. Indeed, each of the 16 created models was applied to classify features of test data so that each epoch lying in the start or rest classes was associated with an epoch prediction of either 0 (indicating nothing is happening) or 1 (indicating a detection). Then, the epoch predictions were grouped appropriately into the corresponding class in which they belonged (resulting in groups of 16 for 4 s windows and groups of 6 for 2 s windows), and a majority vote algorithm (MVA) was used for each group to produce a single prediction (either 0 or 1) per group. If the output of the MVA was a tie, the output of the classifier for that group was 0. Then, that outcome was compared with the real value of the class (0 for the rest class and 1 for the start class). This process can be seen in Figure 5A.

For the pseudo-online analysis, the first 10 trials were used to create the model and the other 6 to test it. For each epoch, the classifier took a decision (an epoch prediction). Afterward, a voting queue algorithm was used. This algorithm determined that a pedaling initiation detection was produced after 5 consecutive detections. The detection was then checked to see if it lied in a start class window, in which case it was a true detection, and otherwise was labeled as a false positive. If many true detections occurred in a fixed start class window, only one true detection was counted. Figure 5B shows this classification analysis.

### 2.4. Post-processing

In order to quantify the results, different parameters were calculated:

- True positive rate (TPR): indicates the percentage of real pedaling intentions that were correctly classified as such,
  (1) $$\begin{array}{r} \text{TPR}=\frac{\text{Number of true detections}}{\text{Number of true events}}. \end{array}$$
- False positives per minute (FP/min): represents how many times per minute the classifier detects a pedaling intent during resting,
  (2) $$\begin{array}{r} \text{FP/min}=\frac{\text{Number of false detections}}{\text{Rest time in minutes}}. \end{array}$$
- Accuracy (Acc): denotes how many pedaling intentions detected were actually real pedaling intentions,
  (3) $$\begin{array}{r} \text{Acc}=\frac{\text{Number of true detections}}{\text{Number of total detections}}. \end{array}$$

It is extremely convenient to have full knowledge of all three parameters. Indeed, if one of the parameters is not known, there are cases where one cannot determine with certainty whether the classifier is working properly or not. For instance, if Acc is 100%, FP/min is 0 and the TPR is missing, it could be because the classifier correctly detected just one pedaling intention out of five, meaning the BCI would not be working very well despite the known parameters having seemingly desirable values.

Moreover, as a unifying metric to differentiate results coming from distinct processing schemes, a new combined parameter was defined. It is called the weighted discriminator (WD) and is a linear combination of TPR, Acc, and the false positive rate (FPR), which is related to FP/min. The weights are chosen to reflect the preferences of the authors in the corresponding parameter. This unified parameter greatly facilitates statistical analysis and comparison between different electrode configurations and feature extraction algorithms. The weights chosen were -1.0 for FPR, which one would want to minimize as much as possible (the authors think false positives are highly undesirable in real-time applications of the BCI); 0.6 for Acc, which the authors want to give slight preference over TPR; and 0.4 for TPR, such that the range of WD goes from -1 (in the worst case scenario) to 1 (in the best case scenario). The equation for WD is then,

(4) $$\begin{array}{r} \text{WD}=0.4\times \text{TPR}+0.6\times \text{Acc}-1.0\times \text{FPR}. \end{array}$$

Here, the false positive rate (FPR) is defined as

(5) $$\begin{array}{r} \text{FPR}=\text{(FP/min)}\times \text{(Duration of a single FP in mins)}, \end{array}$$

where that duration is precisely the window length (either 2 or 4 s) for the offline analysis and 1 s (equivalent to 5 consecutive detections, each represented by the shift of 200 ms) for the pseudo-online analysis.

## 3. Results and discussion

For each subject, the data was processed using both offline and pseudo-online analyses, with two different types of processing windows, five different extraction algorithms, and eight different electrode configurations, for a total of 160 possible combinations per subject. For each combination, the TPR, FP/min and Acc were calculated as in Equations (1–3), and from these, the values of FPR and WD were also computed as indicated in Equations (4) and (5).

### 3.1. Offline analysis

Table 1 shows the values of WD per subject after using an offline analysis of the EEG data. As mentioned before, these were averages that resulted from the cross-validation among the 16 trials (see Section 2.3.6). Additionally, for each subject, type of processing window, and algorithm, the WD was averaged among the eight different electrode configurations (E.C.), and this is referred to here as the E.C.-averaged WD. These were reported in Table 1 as well. Then, based on these E.C.-averaged WD values, the “best” two algorithms (for each subject and type of processing window) were chosen as those corresponding to the two highest averages. The selected algorithms were marked with two asterisks in Table 1.

**Table 1:** WD value for all subjects using an offline analysis.

**S**	**E. C.**	**Offline** −**2 s to 0 s**					**Offline** −**2 s to 2 s**				
		**A**	**B**	**C**	**D**	**E**	**A**	**B**	**C**	**D**	**E**
1	1	0.11	0.09	0.47	0.08	0.40	0.28	0.16	0.60	0.51	0.27
	2	0.21	0.02	0.26	0.17	0.22	0.32	0.02	0.41	0.53	0.47
	3	0.07	0.26	0.40	0.24	0.26	0.17	0.27	0.61	0.50	0.39
	4	0.04	0.01	0.24	0.39	0.14	0.22	−0.05	0.61	0.68^*^	0.57
	5	0.05	0.19	0.30	0.23	0.39	0.10	0.07	0.50	0.33	0.35
	6	0.02	0.06	0.04	0.14	0.18	0.18	0.07	0.28	0.43	−0.01
	7	0.33	0.22	0.10	0.16	0.17	0.14	0.12	0.33	0.38	0.13
	8	0.12	0.16	0.31	0.21	0.16	0.24	−0.12	0.56	0.52	0.36
	Avg.	0.12	0.13	0.27^**^	0.20	0.24^**^	0.21	0.07	0.49^**^	0.48^**^	0.32
2	1	−0.13	0.12	−0.14	−0.07	0.29	0.36	0.68	0.62	0.74	0.72
	2	0.03	0.15	−0.07	0.04	0.41	0.07	0.59	0.39	0.70	0.70
	3	−0.21	0.11	−0.23	−0.04	0.16	0.27	0.71	0.57	0.69	0.67
	4	−0.02	0.12	0.09	−0.11	0.34	0.32	0.48	0.25	0.64	0.71
	5	−0.09	0.16	−0.07	0.09	0.21	0.28	0.68	0.64	0.76^*^	0.74
	6	−0.14	0.34	−0.11	−0.15	0.41	−0.16	0.56	0.58	0.74	0.75^*^
	7	−0.16	0.25	−0.27	−0.09	0.36	0.27	0.66	0.57	0.72	0.76^*^
	8	−0.17	0.41	−0.09	−0.12	0.35	0.29	0.68	0.56	0.75^*^	0.76^*^
	Avg.	−0.11	0.21^**^	−0.11	−0.06	0.32^**^	0.21	0.63	0.52	0.72^**^	0.73^**^
3	1	0.25	0.15	0.20	0.21	0.28	0.47	0.20	0.51	0.61^*^	0.41
	2	0.00	−0.01	0.17	−0.01	0.31	0.30	−0.12	0.51	0.57	0.40
	3	0.15	0.19	0.29	0.07	0.26	0.36	0.13	0.57	0.52	0.41
	4	0.17	0.37	0.19	0.21	0.23	0.30	0.11	0.57	0.55	0.50
	5	0.04	0.26	0.14	0.07	0.30	0.31	0.32	0.58	0.46	0.46
	6	0.02	0.10	0.20	0.19	0.15	0.28	0.07	0.58	0.59	0.27
	7	0.06	0.32	0.20	0.10	0.23	0.21	0.06	0.44	0.57	0.35
	8	−0.15	0.19	0.19	−0.05	0.12	0.29	0.11	0.53	0.57	0.44
	Avg.	0.07	0.20^**^	0.20	0.10	0.24^**^	0.32	0.11	0.54^**^	0.56^**^	0.41
4	1	0.14	0.02	0.47	0.26	0.22	0.35	0.22	0.60^*^	0.61^*^	0.50
	2	−0.03	0.12	0.26	0.32	0.08	−0.01	0.16	0.41	0.48	0.22
	3	0.10	−0.01	0.40	0.42	0.29	0.25	0.27	0.61^*^	0.59	0.53
	4	0.08	0.14	0.24	0.22	0.18	0.14	−0.12	0.61^*^	0.50	0.30
	5	0.13	0.16	0.30	0.34	0.20	0.16	0.21	0.50	0.53	0.38
	6	0.12	0.03	0.04	0.21	0.13	0.10	0.19	0.28	0.57	0.33
	7	0.12	0.24	0.10	0.34	0.16	0.21	0.12	0.33	0.57	0.49
	8	0.06	0.13	0.01	0.43	0.20	0.14	0.21	0.36	0.61^*^	0.40
	Avg.	0.09	0.10	0.23^**^	0.32^**^	0.18	0.17	0.16	0.46^**^	0.56^**^	0.39
5	1	0.17	0.18	0.25	0.27	0.10	0.59^*^	0.25	0.58	0.54	0.52
	2	0.11	0.02	0.07	0.15	0.08	0.53	0.15	0.32	0.56	0.40
	3	0.06	0.10	0.17	0.24	0.12	0.43	0.16	0.33	0.53	0.31
	4	−0.02	−0.08	0.01	−0.06	−0.02	0.48	0.23	0.10	0.24	0.31
	5	0.26	0.21	0.11	0.13	0.05	0.49	0.11	0.32	0.47	0.42
	6	0.24	0.03	0.26	0.27	0.09	0.42	0.28	0.45	0.50	0.46
	7	0.33	0.00	0.26	0.20	0.03	0.54	0.09	0.55	0.53	0.37
	8	0.15	-0.11	0.20	0.26	0.20	0.50	0.32	0.47	0.55	0.41
	Avg.	0.16	0.05	0.17^**^	0.18^**^	0.08	0.50^**^	0.20	0.39	0.49^**^	0.40

*Results for the 2 s processing windows are shown on the left, while those of the 4 s processing windows are shown on the right. The best two feature extraction algorithms for each subject (S) are indicated with two asterisks. The best electrode configurations for those algorithms are also pointed out with one asterisk (those within 0.01 of the highest WD value among those two-asterisk-columns)*.

The 4 s processing windows include time after which the movement has initiated (see Figure 3) and are comprised of 16 epochs, while the 2 s processing windows only include the 6 epochs preceding movement initiation. Thus, intuitively, it is much more demanding to detect the ERD/ERS phenomena when using the 2 s windows than with the 4 s windows. Indeed, as expected, the WD values for the 2 s processing windows were considerably lower (the E.C.-averaged WD values did not go beyond 0.32) than those of the 4 s windows (where an E.C.-averaged WD value of 0.73 was observed).

Clearly, the results varied across subjects and it was evident that some electrode configurations and algorithms were better suited to certain individuals. With this in mind, the goal was to find the “best” combination of feature extraction algorithm and electrode configuration for each person. This selection was restricted to the 4 s processing windows given the stark difference in the quality of the results when compared to the 2 s windows. The “best” BCI for each subject was determined using the following procedure:

1. The best two algorithms were selected based on the two highest E.C.-averaged WD values. These were marked with double asterisks in Table 1.
2. Among the 16 WD values associated to the two algorithms and eight electrode configurations, the maximum WD value was preselected along with all those WD values within 0.01 of that maximum. These were marked with a single asterisk in Table 1.
3. Lastly, among the preselected combinations, the one corresponding to the electrode configuration with the lowest number of electrodes was chosen (see Figure 4). If there was a tie on the number of electrodes, the one with the highest WD was selected. If the tie continued, one was chosen at random.

The best personalized BCIs with an offline analysis and 4 s processing windows were tabulated in Table 2. The cross-validation averages and standard deviations of the TPR, FP/min and Acc were reported.

**Table 2:** TPR, FP/min and Acc results for the best offline feature extraction algorithm and electrode configuration of each subject (S) with 4 s processing windows.

**S**	**Algorithm**	**E.C.**	**TPR (%)**	**FP/min**	**Acc (%)**
1	D	4	63.8 ± 24.5	1.69 ± 2.18	89.1 ± 13.7
2	E	6	50.0 ± 27.3	0.19 ± 0.75	93.8 ± 25.0
3	D	1	71.3 ± 29.2	2.25 ± 2.57	78.6 ± 26.9
4	C	3	67.5 ± 20.5	2.44 ± 2.50	83.7 ± 16.8
5	A	1	82.5 ± 21.8	3.56 ± 4.55	82.8 ± 20.8
		Average	67.0	2.03	85.6

### 3.2. Pseudo-online analysis

A more detailed examination of the pseudo-online analysis is presented next. This is due to the long-term goal of using these BCIs in active therapies to aid recovering stroke patients, where an online processing of the signals is crucial. In real-time applications it would be ideal to use the 2 s windows over the 4 s windows, since they would produce more natural movement in a patient. Having said that, this choice is contingent upon the quality of the results. For the pseudo-online analysis, the test data was processed differently using the last 6 trials and then averaged (with the first 10 training trials being used to create the models). In fact, computing the WD values analogously to Table 1 again showed much better results for 4 s processing windows than with 2 s windows. Thus, the results suggest that it is preferable to use the 4 s processing windows, even if slightly delayed movement is produced as a result of this choice. Indeed, from now on, only the 4 s processing windows will be investigated for the pseudo-online analysis.

#### 3.2.1. Best personalized BCIs

WD values were tabulated as in Table 1, but for compactness, only the WD results for the two best algorithms of each subject using 4 s processing windows were shown in Table 3. Note that the best two algorithms per subject were the same for the offline processing as with the pseudo-online processing, but this is a consequence of this particular data set. In general, the best two algorithms could be completely different for the two types of analyses.

**Table 3:** WD values for the best two feature extraction algorithms of each subject with pseudo-online analysis for the 4 s processing windows.

**E. C.**	**Subject 1**		**Subject 2**		**Subject 3**		**Subject 4**		**Subject 5**	
	**C**	**D**	**B**	**E**	**C**	**D**	**C**	**D**	**A**	**E**
1	0.24	−0.04	0.81	0.81	0.42^*^	0.37	0.74^*^	0.47	0.34	0.36
2	0.22	0.18	0.69	0.77	0.33	0.43^*^	0.74^*^	0.48	0.35	0.42^*^
3	0.30^*^	0.15	0.61	0.84	0.41	0.29	0.67	0.51	0.32	0.24
4	0.29	0.31^*^	0.53	0.85	0.38	0.38	0.67	0.38	0.23	0.42^*^
5	0.22	0.11	0.60	0.82	0.36	0.29	0.67	0.38	0.35	0.27
6	0.16	0.27	0.73	0.77	0.37	0.33	0.68	0.57	0.35	0.23
7	0.17	0.14	0.86	0.84	0.28	0.31	0.71	0.58	0.29	0.24
8	0.23	0.06	0.89^*^	0.76	0.35	0.35	0.70	0.57	0.28	0.32

*The best electrode configurations are pointed out with one asterisk*.

Also, notice the apparent contradiction that WD results for some subjects seem to be higher with the pseudo-online analysis than with the offline analysis. This happened due to the way the false positives were detected, and thus in the way FP/min and FPR were computed with the two different analyses (see 5). Indeed, due to the nature of the offline analysis, only one FP can be detected per rest window. This means that if the FPs were abundant in pseudo-online (say, 6 per rest window), the FPR would be underestimated in the offline analysis, but if the FPs were scarcer (an average of less than 4 per 4 s rest window), the offline analysis would overestimate the FPR and produce lower WD offline values. This can be observed when computing the FPR from Tables 2, 4 and comparing. Thus, it is not wise to compare results between offline and pseudo-online analysis via WD. However, given a fixed type of analysis, it would be justifiable to compare between electrode configurations and feature extraction algorithms.

**Table 4:** TPR, FP/min, and Acc results for the best pseudo-online feature extraction algorithm and electrode configuration of each subject (S) with 4 s processing windows.

**S**	**Algorithm**	**E.C.**	**TPR (%)**	**FP/min**	**Acc (%)**
1	D	4	83.3 ± 15.1	10.3 ± 6.33	25.1 ± 11.7
2	B	8	76.7 ± 23.4	0.08 ± 0.20	96.7 ± 8.16
3	D	2	86.7 ± 20.7	6.83 ± 3.29	32.1 ± 9.33
4	C	2	76.7 ± 15.1	3.74 ± 2.20	81.8 ± 15.9
5	E	4	60.0 ± 17.9	3.73 ± 1.82	39.9 ± 14.7
		Average	76.7	4.94	55.1

The best personalized BCIs were selected as described in Section 3.1. The TPR, FP/min and Acc for those personalized configurations are displayed in Table 4, where the standard deviations were computed from the 6 different test trials of each subject.

#### 3.2.2. Are personalized BCIs worthwhile?

The purpose of this section is to show that the use of personalized BCIs is indeed preferable over a more traditional approach, were the electrode configuration and feature extraction algorithm are fixed and are used uniformly for all subjects. The goal is also to determine to what extent the personalization of the BCI among all subjects makes sense for this data set. To do this, a criteria to find the best feature extraction algorithm among all subjects (not *per* subject) is described. Then, for that fixed algorithm a procedure to choose the best (personalized) electrode configuration per subject is outlined. Next, the results are statistically compared with those of the “fully” personalized BCI described in the previous section (reported in Table 4). Finally, given the choice of the best algorithm, a method to choose the best electrode configuration among all subjects is described, leading to a fixed algorithm and electrode configuration. Again, the results are statistically compared with those of the “fully” personalized BCI.

The procedure used to choose the best uniform feature extraction algorithm was as follows:

1. For each subject and algorithm its E.C.-averaged WD was computed (WD values were averaged among electrode configurations). These values were then averaged among the subjects, so that a single averaged WD represented each algorithm.
2. The algorithm associated to the highest such average was selected.

Taking these steps yielded that the best uniform algorithm was D, followed closely by algorithms C and E. Next, given this choice of best algorithm, the method to select the best electrode configuration per subject was:

1. Given the best uniform algorithm, there are eight WD values associated to the eight electrode configurations per subject. The maximum WD value was preselected along with all those WD values within 0.01 of that maximum.
2. Among the preselected WD values, the one corresponding to the electrode configuration with the lowest number of electrodes was chosen (see Figure 4). If there was a tie on the number of electrodes, the one with the highest WD was selected. If the tie continued, one was chosen at random.

For a pseudo-online analysis and 4 s processing windows, the BCIs associated with the best uniform algorithm and a personalized electrode configuration are presented in Table 5.

**Table 5:** TPR, FP/min and Acc results for the pseudo-online feature extraction algorithm D and the best personalized electrode configuration of each subject (S) with 4 s processing windows.

**S**	**E.C.**	**TPR (%)**	**FP/min**	**Acc (%)**
1	4	83.3 ± 15.1	10.3 ± 6.33	25.1 ± 11.7
2	4	66.7 ± 16.3	0.26 ± 0.45	90.0 ± 16.7
3	2	86.7 ± 20.7	6.83 ± 3.29	32.1 ± 9.33
4	6	86.7 ± 16.3	3.76 ± 2.10	47.0 ± 9.38
5	1	56.7 ± 23.4	3.83 ± 1.07	35.5 ± 9.63
	Average	76.0	5.00	46.0

To get a better statistical sample to compare the results of Tables 4, 5, the WD values of each of the 6 test trial sessions were computed for each subject. This yielded 30 WD values (from 5 subjects and 6 trial sessions) associated to the BCIs in Table 4 and the same with Table 5. A Wilcoxon signed-rank test determined that the differences of these samples were not statistically significant (*p* = 0.07) at the standard 5% significance level. Thus, for this particular set of subjects it would make sense to use D as a fixed feature extraction algorithm. This can be helpful to reduce processing time when determining the best possible personalized BCI, since one must only seek between different electrode configurations, instead of different algorithms plus electrode configurations.

Lastly, the idea was to find the best “traditional” approach by finding the feature extraction algorithm and electrode configuration that best suited *all* subjects. The procedure to find the algorithm that best fitted all subjects was already described, while that of finding the optimal electrode configuration was as follows:

1. Given the best uniform algorithm, the WD values were averaged among the subjects for each electrode configuration. This gave a single averaged WD for each electrode configuration.
2. The electrode configuration associated to the highest such average was selected.

Following this procedure yielded that the “traditional” approach that best suited all subjects was that associated to the fixed use of algorithm D and electrode configuration 4. The results associated to these parameters are shown in Table 6.

**Table 6:** TPR, FP/min, and Acc results for the pseudo-online feature extraction algorithm D and electrode configuration 4 for each subject (S) with 4 s processing windows.

**S**	**TPR (%)**	**FP/min**	**Acc (%)**
1	83.3 ± 15.1	10.3 ± 6.33	25.1 ± 11.7
2	66.7 ± 16.3	0.27 ± 0.45	90.0 ± 16.7
3	96.7 ± 8.16	10.1 ± 4.06	26.3 ± 7.89
4	93.3 ± 10.3	9.50 ± 2.51	27.5 ± 7.85
5	70.0 ± 21.0	12.6 ± 3.32	17.2 ± 3.17
Average	82.0	8.56	37.2

The results of Tables 4, 6 were compared using the Wilcoxon signed-rank test as described previously. In this case, there were statistically significant differences (*p* = 0.0007) of the two samples. A quick inspection of Table 6 shows the results are much less preferable than those of Table 4. Thus, it is clear that a personalized BCI seems to be more suitable than a traditional approach. This shows the power of considering personalized BCIs, as they give greater versatility in providing the best results possible for a particular individual.

### 3.3. Further discussion

To summarize, as one would expect, the 2 s processing windows with only time before movement onset produce results of lower quality (in terms of WD) than the 4 s processing windows which include time before and after the movement onset. For this reason, it seems preferable to use 4 s processing windows for most purposes, even if they may cause a slight delay when used in real-time applications. Furthermore, based on the WD values, a rigorous procedure to select the best feature extraction algorithm and electrode configuration for each subject was described. Generally speaking, the optimal combination involves high WD values along with the lowest number of electrodes possible. The “minimization” of the number of electrodes is in part to qualitatively pinpoint the areas of the brain that produce the best results, and in part because results can be processed faster (which may be useful in real-time processing of signals). Lastly, statistical analyses determined that the fully personalized BCIs produced better results than a traditional approach. Naturally, other choices of feature extraction algorithms and electrode configurations than those provided in this work are possible, and those are left to the criteria of new researchers. However, the overarching philosophy of personalizing the BCI to each subject is the key point.

Regarding other work in the literature, it should be mentioned that most studies are analyzed offline (Bai et al., 2007, 2011; Lew et al., 2012; Bulea et al., 2014; Jiang et al., 2015; Sburlea et al., 2015). The results seem comparable in terms of TPR and Acc. Having said that, in many cases comparisons are difficult, since either the relevant parameters are not reported or they must be post-processed from their own results. One study with a pseudo-online analysis Ibáñez et al. (2010) also presented similar results in all parameters. Thus, the results in this work seem to be consistent with those in the literature.

Pseudo-online personalized BCI models were preferred in comparison with offline models, due to their potential use with exoskeletons and other real-time applications. Specifically, the goal is to use this approach in therapies to improve the pedaling capabilities of recovering stroke patients who will be aided by an exoskeleton (or some other medium). Indeed, pedaling seems to be a natural intermediate step before fully rehabilitating gait. The results in this work are valid for the small sample of healthy subjects studied, but to make any assertions in terms of the quality of the results in larger population samples of healthy or recovering patients, more experiments are needed. Having said that, when compared with a traditional approach, the idea of personalizing the BCIs is also expected to yield better results in the population of stroke patients.

Regarding the limitations, a potential for concern in the results of this work is the relatively high number of FP/min which is highly undesirable when trying to detect self-initiated movement. Thus, there is still room for improvement in trying to reduce this variable. Finally, another future avenue of research with these personalized BCI models is the detection of pedaling or walking initiation via motor imagery, for which new protocols must be designed.

## 4. Conclusions

A personalized BCI model aimed at the prediction of the intention of self-initiated pedaling was successfully designed for each subject. This included choosing among different types of processing windows (2 possible), feature extraction algorithms (5 possible) and electrode configurations (8 possible). Moreover, this process was done both for offline analysis and pseudo-online analysis. More precisely, a metric developed by the authors, called the weighted discriminator (WD), was used to compare different BCIs. Indeed, a procedure based on the WD and used to select the best personalized BCI model of each individual was described in detail. This procedure resulted in a desirable choice of feature extraction algorithm and electrode configuration for each specific subject. In general, a processing window of longer duration and including time after movement onset was preferred over the other type of processing window. The results in pseudo-online for the best BCI models of each subject were in average 76.7% of TPR, 4.94 FP/min and 55.1% of Acc. More importantly, statistical analyses were used in a systematic fashion to show that personalized BCIs provide better results than a traditional approach, where a subject-independent feature extraction algorithm and electrode configuration are used for all subjects. Finally, in the context of the rehabilitation of stroke patients, this approach may be useful in active therapies to interface with a lower limb exoskeleton.

## Author contributions

MR is responsible for the design, implementation, acquisition and data analysis. MR and EI took part in the interpretation of data. In addition, EI and MO supervised the work and contributed with the revision process. JA actively contributed as director of the work.

### Conflict of interest statement

The authors declare that the research was conducted in the absence of any commercial or financial relationships that could be construed as a potential conflict of interest.

## Abbreviations

E.C.: Electrode Configuration
MVA: Majority Vote Algorithm
Acc: Accuracy
WD: Weighted Discriminator.
